# Modulation-Prior Enhanced Cross-Modal Graph Learning for Low-SNR Automatic Modulation Recognition

**DOI:** 10.3390/s26185888

**Published:** 2026-09-17

**Authors:** Haibo Su, Yixiang Luomei, Zhentao Yang, Feiyu Mou, Zhenghong Lu, Yi Chen

**Affiliations:** College of Future Information Technology, Fudan University, Shanghai 200433, China; hbsu25@m.fudan.edu.cn (H.S.); 24110720175@m.fudan.edu.cn (Z.Y.); 25213090177@m.fudan.edu.cn (F.M.); 25213090165@m.fudan.edu.cn (Z.L.); 25213090073@m.fudan.edu.cn (Y.C.)

**Keywords:** automatic modulation recognition, low-SNR signal recognition, cross-modal consistency learning, multi-resolution short-time Fourier transform, graph neural network

## Abstract

Automatic modulation recognition (AMR) is a key enabling technique for intelligent spectrum sensing, non-cooperative wireless signal analysis, and communication monitoring. However, its reliability degrades significantly under low signal-to-noise ratio (SNR) conditions, where noise weakens local I/Q waveform patterns, blurs time-frequency structures, and limits the robustness of single-domain representations. Although multimodal AMR methods have improved recognition by combining I/Q and time-frequency representations, many of them still use fixed fusion rules, which may not reflect the changing reliability of different modalities across SNR levels. In addition, most existing backbones model signal sequences in a Euclidean manner and lack an explicit mechanism to capture adaptive relational structures among signal tokens under strong noise. To address these limitations, this paper proposes MPCG-Net, a modulation-prior-enhanced cross-modal graph learning framework for low-SNR AMR. MPCG-Net first performs modulation-prior-enhanced cross-modal encoding, where learnable multi-resolution spectro-temporal representation learning enhances the input representation and training-stage SNR-aware cross-modal consistency alignment improves the complementary interaction between I/Q and spectro-temporal tokens. A modulation context memory module then refines the fused tokens before graph construction. In the graph-based relational learning stage, a temporal similarity graph with self-loops, local temporal-chain edges, and top-*k* feature-similarity edges is built to learn more discriminative graph-level signal representations. Experiments on RadioML2016.10A, RadioML2016.10B, and RML22 validate the effectiveness of the proposed design. Compared with the strongest baseline on each dataset, MPCG-Net improves the average accuracy in the −20 to 0 dB low-SNR range by 6.90, 6.45, and 1.66 percentage points on RadioML2016.10A, RadioML2016.10B, and RML22, respectively, while maintaining a lightweight model scale.

## 1. Introduction

Automatic modulation recognition (AMR) aims to identify the modulation format of a received signal without prior knowledge of transmitter-side parameters. As a key intermediate stage between signal detection and demodulation, AMR provides essential prior information for intelligent spectrum sensing, cognitive radio, non-cooperative communication analysis, electronic surveillance, spectrum management, and communication monitoring [1,2,3,4]. In practical wireless systems, the receiver may not know the carrier frequency offset, symbol timing, channel state, transmitter behavior, or interference condition in advance. Therefore, reliable modulation recognition is important for blind receiver design, adaptive waveform selection, dynamic spectrum access, and radio environment awareness [2,5,6]. With the increasing complexity of electromagnetic environments and the growing demand for intelligent wireless systems, robust AMR has become a fundamental problem in both classical signal processing and data-driven wireless intelligence [7,8,9].

Early AMR methods can generally be divided into likelihood-based and feature-based approaches [1,2,10]. Likelihood-based methods formulate modulation recognition as a statistical hypothesis testing problem. The receiver evaluates the likelihood of the observed signal under different candidate modulation hypotheses and then selects the modulation type that maximizes the likelihood function [10,11,12]. When the signal model, noise distribution, synchronization information, and channel parameters are accurately known, likelihood-based methods can provide strong theoretical performance. However, their practical deployment is often limited by high computational complexity and the need to estimate nuisance parameters such as phase offset, frequency offset, timing error, channel gain, and noise variance [10,11]. In non-cooperative scenarios, these assumptions are usually difficult to satisfy, causing performance degradation and implementation difficulty.

Feature-based methods provide another important traditional AMR paradigm. These methods first extract manually designed signal features and then use decision rules or statistical classifiers for modulation classification [1,2]. Representative features include instantaneous amplitude, phase, and frequency statistics; higher-order moments and cumulants; cyclic spectral features; wavelet-domain descriptors; constellation-based features; and time-frequency representations [2,13,14,15,16,17]. For example, higher-order cumulants can exploit statistical differences among modulation formats, while cyclostationary features capture periodic structures caused by symbol rates, carrier frequencies, or modulation mechanisms [13,15]. These handcrafted features are interpretable and effective in controlled conditions. Nevertheless, their performance is sensitive to noise, fading, frequency offset, sampling mismatch, and short observation length. In particular, under low signal-to-noise ratio (SNR) conditions, manually designed features may lose discriminative information, and feature selection becomes highly dependent on expert knowledge and signal assumptions [1,2,18].

To improve the flexibility of traditional feature-based pipelines, machine learning methods have been introduced into AMR. Support vector machines, k-nearest neighbor classifiers, decision trees, random forests, and shallow neural networks have been used to learn nonlinear decision boundaries from handcrafted features [18,19,20,21,22]. Compared with fixed threshold-based classifiers, these methods can better adapt to complex feature distributions. However, their performance still largely depends on the quality and robustness of the input features. Once the extracted features become unreliable under low SNR or channel mismatch, the classifier itself has limited ability to recover the lost modulation information. Therefore, although traditional machine learning improves classification flexibility, it does not fundamentally eliminate the dependence on handcrafted feature engineering [7,8,9].

Deep learning has significantly changed the development of AMR by enabling end-to-end representation learning from raw I/Q samples or transformed signal representations [7,8,9]. O’Shea et al. first demonstrated that convolutional neural networks can be adapted to complex temporal radio signals and can outperform expert-feature-based methods in modulation classification tasks [23]. West and O’Shea further investigated deeper neural architectures, including convolutional and recurrent structures, showing the potential of deep models for learned wireless signal representations [24]. Subsequent studies explored CNNs, CLDNNs, residual networks, recurrent neural networks, long short-term memory networks, gated recurrent units, attention mechanisms, and Transformer-based architectures for AMR [25,26,27,28,29,30,31,32]. Among these methods, MCLDNN learns spatiotemporal multi-channel features from I/Q signals, AMCNet improves low-SNR recognition by combining frequency-domain denoising with multi-scale feature extraction, and FEA-T introduces frame-wise embedding aided Transformer modeling to capture global correlation features [33,34,35]. These methods improve modulation recognition by learning hierarchical temporal, spatial, or attention-based signal representations. However, many deep AMR models still process signals as Euclidean sequences or images, mainly emphasizing local convolutional patterns, sequential dependencies, or global attention responses. Under strong noise, useful modulation evidence may appear sparsely and irregularly across temporal segments or transformed feature domains, making it difficult for purely sequential or image-based backbones to explicitly capture adaptive relational structures among signal tokens.

Another important trend is multimodal or multidomain AMR. Different signal representations contain complementary information. Raw I/Q samples preserve fine-grained waveform evolution in the time domain, while time-frequency representations, constellation diagrams, amplitude-phase features, frequency-domain features, and cyclostationary features reveal different aspects of modulation behavior [36,37,38,39,40]. Recent studies have shown that combining multiple modalities can improve recognition performance compared with single-domain input representations [36,37,38,39,40,41,42]. For example, AbFTNet aligns I/Q and fractional Fourier representations before cross-modal aggregation and introduces contrastive predictive coding as auxiliary supervision [41]. IQFormer combines I/Q and STFT representations through convolutional fusion, followed by recurrent and attention-based feature encoding [43]. These methods demonstrate the benefit of heterogeneous signal views, but two issues remain relevant to low-SNR AMR. First, low-SNR observations contain limited information about modulation-related signal parameters, while a single fixed-resolution transformation may further form an unfavorable information bottleneck. Together with the intrinsic time–frequency resolution tradeoff of the STFT [44], this makes it difficult for one analysis scale to expose both short-duration waveform variations and stable spectral structures to the subsequent network. Second, multimodal fusion alone does not guarantee reliable local token representations, because modulation evidence corrupted by strong noise may remain fragmented and should be refined using its surrounding signal context before subsequent relational modeling.

Graph neural networks (GNNs) provide a promising way to model non-Euclidean relational structures by representing entities as nodes and their dependencies as edges [45,46,47,48,49]. In signal recognition tasks, graph-based modeling can treat signal segments or feature tokens as graph nodes and use edges to describe temporal adjacency, feature similarity, learned correlations, or domain-specific relationships. Graph convolution, graph attention, and graph pooling mechanisms can then be used to propagate information and learn graph-level representations [45,46,47,48,49,50]. In graph-based AMR, AvgNet constructs adaptive visibility graphs directly from the I/Q sampling sequences and performs DiffPool-based classification [51]. CTGNet divides I/Q sequences into local windows, estimates adjacency through Transformer attention, and combines GraphSAGE with graph pooling [52]. STF-GCN embeds spatial, temporal, and frequency-domain information into graph nodes and estimates graph connectivity through adaptive correlation [53]. Although these methods improve signal-to-graph mapping, their graph construction is still organized around sampling-level or locally segmented features. Under low-SNR conditions, constructing relations before sufficiently refining noisy multimodal evidence may produce unstable neighborhood information, while non-local dependencies among context-refined multimodal tokens remain underexplored. Table 1 summarizes representative multimodal and graph-based AMR methods in terms of input representation, fusion strategy, auxiliary supervision, graph construction, and training objective.

To address the above limitations, this paper proposes MPCG-Net, whose processing flow consists of two connected parts. The first part focuses on forming reliable signal representations before graph construction. The received sequence is encoded through complementary I/Q and spectro-temporal branches, and multiple STFT resolutions are adaptively combined because modulation evidence may appear at different temporal and spectral scales. After cross-modal fusion, the modulation context memory module further refines each token using its surrounding observations, reflecting the temporal dependence introduced by pulse shaping and continuous phase evolution. Cross-modal consistency learning further regularizes the two views during training and promotes their complementary interaction. The second part performs graph-based relational learning over the refined multimodal tokens. A temporal similarity graph preserves local temporal continuity while adding data-dependent non-local connections, and hierarchical graph propagation produces the final graph-level representation. In this way, modulation-domain knowledge first guides the formation of robust node features, after which graph learning organizes their local and non-local dependencies for classification.

The main contributions of this paper are summarized as follows:

(1) A modulation-prior-enhanced cross-modal graph learning framework, named MPCG-Net, is proposed to improve low-SNR AMR performance. By analyzing common modulation signal models, we summarize two structural priors that remain informative under strong noise: a spectro-temporal scale prior associated with modulation evidence distributed across different time–frequency resolutions, and a temporal-dependence prior induced by pulse shaping and phase evolution. MPCG-Net incorporates these priors into cross-modal representation learning and then models relations among the refined tokens through graph learning.
(2) The spectro-temporal scale prior is implemented through a learnable multi-resolution STFT encoder, which uses complementary analysis windows to represent the unavoidable time–frequency resolution tradeoff. Cross-modal consistency learning serves as an auxiliary training regularizer that promotes complementary information exchange and representation agreement between the I/Q and spectro-temporal branches under varying SNR conditions.
(3) The temporal-dependence prior is implemented through a modulation context memory module that refines fused tokens using surrounding signal evolution before graph construction. The subsequent temporal similarity graph preserves short-range temporal continuity while selecting data-dependent top-*k* feature-similarity edges over the full token sequence. In contrast to candidate relations constrained by a fixed local scanning range, this construction enables non-adjacent tokens carrying similar modulation evidence to interact during hierarchical graph learning.
(4) Extensive experiments demonstrate that MPCG-Net achieves superior low-SNR recognition performance within a lightweight parameter range. Compared with the strongest baseline on each dataset, MPCG-Net improves the average accuracy in the −20 to 0 dB range by 6.90, 6.45, and 1.66 percentage points on RadioML2016.10A, RadioML2016.10B, and RML22, respectively, indicating that the gain does not depend on a substantially larger model capacity.

The remainder of this paper is organized as follows. Section 2 presents the signal model and problem formulation, including the received baseband signal representation, AMR task definition, and low-SNR recognition setting. Section 3 details the proposed MPCG-Net framework, including modulation-prior-enhanced cross-modal representation learning and graph-based relational learning. Section 4 evaluates MPCG-Net on multiple benchmark datasets through baseline comparisons, ablation studies, and low-SNR performance analysis. Finally, Section 5 discusses the main findings and concludes this paper.

## 2. Signal Model and Problem Formulation

### 2.1. Received Baseband Signal Model

Consider a discrete-time complex baseband signal observed by a non-cooperative receiver. For a modulation class y∈Y={1,2,…,C}, a general received sequence can be expressed as

(1) $$r\left[n\right]=e^{j(2\pi \Delta fnT_{s}+\phi )}\left(h_{c}*s_{y}\right)\left(nT_{s}\right)+\eta \left[n\right],\;n=0,1,\ldots ,L-1,$$

where $s_{y}\left(t\right)$ is the transmitted complex envelope, $h_{c}\left(t\right)$ is the channel impulse response, * denotes convolution, $T_{s}$ is the sampling interval, Δf and ϕ denote carrier-frequency and phase offsets, and η[n] is additive noise. For common digital modulation types, the transmitted signal may be represented by the linear-modulation and continuous-phase-modulation (CPM) models [54,55]:

(2) $$s_{y}\left(t\right)=\left\{\begin{array}{cc} \sum_{m}a_{m}p\left(t-mT\right), & y\in Y_{\mathrm{lin}},\\ A\exp\;\left\{j\left[2\pi h\sum_{m}a_{m}q\left(t-mT\right)+\phi_{0}\right]\right\},\; & y\in Y_{\mathrm{cpm}}, \end{array}\right.$$

where $a_{m}$ is a modulation symbol, p(t) is the pulse-shaping response, *T* is the symbol period, *h* is the modulation index, and $q\left(t\right)=\int_{-\infty }^{t}g\left(\tau \right)\;d\tau$ is the phase response associated with the CPM frequency pulse g(t). The first branch covers representative PAM, PSK, and QAM signals, whereas the second describes continuous-phase signals such as CPFSK and GFSK. In blind signal reception, these equations are introduced to reveal structural properties shared by different modulation types. The analog classes in the evaluated datasets also exhibit envelope, phase, and spectro-temporal structure, but the finite-alphabet argument used below is stated specifically for digital modulation.

The input sample is represented by its in-phase and quadrature components,

(3) $$x=\left[\begin{array}{cccc} ℜ\{r[0]\} & ℜ\{r[1]\} & \cdots & ℜ\{r[L-1]\}\\ ℑ\{r[0]\} & ℑ\{r[1]\} & \cdots & ℑ\{r[L-1]\} \end{array}\right]\in R^{2\times L}.$$

In the evaluated RadioML-style benchmarks, L=128 is used unless otherwise specified. The SNR associated with each sample is denoted by *s* and defined as

(4) $$s=10\log_{10}\left(\frac{P_{\mathrm{sig}}}{P_{\mathrm{noise}}}\right),$$

where $P_{\mathrm{sig}}$ and $P_{\mathrm{noise}}$ denote the signal and noise powers, respectively.

### 2.2. Modulation-Prior Formulation

Under strong noise, observations carry less Fisher information about unknown signal parameters, while the Cramér–Rao lower bound on unbiased estimation error correspondingly increases [56]. Structural regularities shared by modulation signals can therefore reduce reliance on unreliable intermediate parameter estimates. From this perspective, Equations (1) and (2) motivate modeling the following two modulation priors:

(5) $$P_{\mathrm{mod}}=\left\{P_{\mathrm{scale}},P_{\mathrm{temporal}}\right\}.$$

*Spectro-temporal scale prior $P_{\mathrm{scale}}$.* The effective temporal and spectral scales of a modulated waveform vary with the symbol period, pulse shape, frequency pulse, and phase trajectory. Moreover, for a window whose temporal and frequency spreads are defined as standard deviations under the Fourier convention $e^{-j2\pi ft}$, STFT analysis obeys [44]

(6) $$\Delta t_{w}\Delta f_{w}\geq \frac{1}{4\pi }.$$

Consequently, a single window cannot provide arbitrarily fine resolution in both domains. The prior therefore states that modulation evidence should be observed at complementary spectro-temporal resolutions; it does not prescribe the specific window lengths or their learned weights.

*Temporal-dependence prior $P_{\mathrm{temporal}}$.* Let $M=T/T_{s}$ and absorb pulse shaping and the channel into an equivalent discrete response $p_{\mathrm{eq}}\left[n\right]$. After suppressing the carrier-frequency and phase offsets for this correlation argument, denote the resulting equivalent observation by $\tilde{r}\left[n\right]$, where $\tilde{r}\left[n\right]=\sum_{k}a_{k}p_{\mathrm{eq}}\left[n-kM\right]+\eta \left[n\right]$. For independent zero-mean symbols with variance $\sigma_{a}^{2}$ and complex white noise with variance $\sigma_{\eta }^{2}$,

(7) $$R_{\tilde{r}}\left[n,\ell \right]=E\;\left\{\tilde{r}\left[n\right]{\tilde{r}}^{*}\left[n-\ell \right]\right\}=\sigma_{a}^{2}\sum_{k}p_{\mathrm{eq}}\left[n-kM\right]p_{\mathrm{eq}}^{*}\left[n-\ell -kM\right]+\sigma_{\eta }^{2}\delta \left[\ell \right].$$

Thus, neighboring samples and their tokens are generally not independent when shifted pulses overlap [57]. CPM provides an additional and explicit form of memory because its current phase accumulates contributions from earlier symbols in Equation (2). The temporal-dependence prior therefore states that a local token should be refined using surrounding observations before pairwise relations are estimated.

MPCG-Net maps $P_{\mathrm{scale}}$ and $P_{\mathrm{temporal}}$ to its multi-resolution spectro-temporal encoder and pre-graph modulation context memory module, respectively, thereby exposing modulation evidence across complementary analysis scales and consolidating temporally dependent evidence before graph construction.

### 2.3. Automatic Modulation Recognition Task

Given an input I/Q segment *x*, AMR aims to learn a mapping

(8) $$F_{\Theta }:x\to \hat{y},$$

where Θ denotes the trainable parameters, *C* is the number of modulation classes, and $\hat{y}$ is the predicted modulation label. For a training set $D={\left\{\left(x_{i},s_{i},y_{i}\right)\right\}}_{i=1}^{N}$, the SNR label $s_{i}$ is used only as auxiliary training information for the cross-modal consistency alignment objective, while the classification mapping itself depends on the received I/Q segment. The main classification objective is formulated as

(9) $$L_{\mathrm{CE}}=-\frac{1}{N}\sum_{i=1}^{N}\sum_{c=1}^{C}I\left(y_{i}=c\right)\log p_{\Theta }\left(c|x_{i}\right),$$

where $p_{\Theta }\left(c|x_{i}\right)$ is the predicted probability of class *c* and I(·) is the indicator function.

### 2.4. Low-SNR Recognition Setting

This work focuses on robust recognition under strong noise. For the two RadioML2016 datasets, namely RadioML2016.10A and RadioML2016.10B, the available SNR range is −20 to 18 dB [58], while the RML22 dataset covers −20 to 20 dB [59]. Following the low-SNR evaluation protocol used in this paper, the low-SNR range is defined as s∈[−20,0] dB. In this range, local I/Q waveform patterns are easily corrupted, and time-frequency structures may also become blurred. Consequently, a reliable AMR model should combine modulation-prior-enhanced cross-modal representation learning with graph-based relational learning rather than depend on a single fixed representation.

Based on this formulation, MPCG-Net maps each received signal into complementary I/Q and spectro-temporal token representations, consolidates temporally dependent modulation evidence, and constructs a graph over the refined tokens for graph-level classification.

## 3. Proposed MPCG-Net Framework

### 3.1. Overall Framework

**Figure 1:**
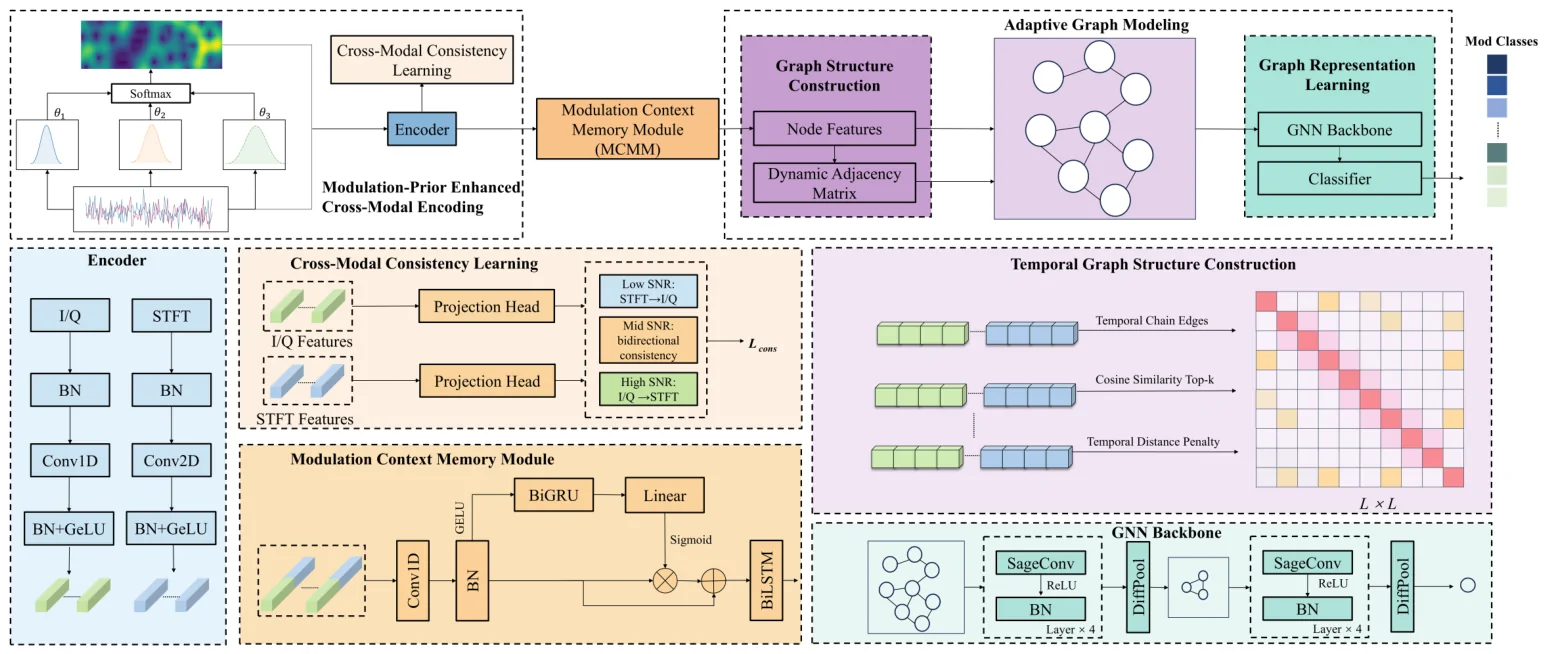
Overall architecture of the proposed MPCG-Net framework. The model first performs modulation-prior-enhanced cross-modal encoding through learnable multi-resolution spectro-temporal representation learning and training-stage SNR-aware cross-modal consistency alignment, then refines the fused tokens with a modulation context memory module, and finally applies graph-based relational learning for low-SNR modulation recognition.

As illustrated in Figure 1, MPCG-Net adopts a progressive dual-branch recognition pipeline for robust low-SNR AMR. The overall framework consists of two connected parts: modulation-prior-enhanced cross-modal representation learning and graph-based relational learning. Starting from the received I/Q samples, the first part extracts time-domain waveform tokens and derives complementary spectro-temporal tokens from the same signal. A learnable multi-resolution spectro-temporal encoder generates adaptive time-frequency features, while cross-modal consistency learning acts as an auxiliary training regularizer that promotes complementary information exchange and representation agreement between the two views. The fused token sequence is then refined by a modulation context memory module. In the second part, a temporal similarity graph is constructed over the refined tokens to produce the final modulation prediction. This design enables MPCG-Net to jointly exploit local waveform structures, adaptive time-frequency cues, temporally dependent modulation evidence, and non-local token dependencies under severe noise conditions.

Following Section 2.2, MPCG-Net introduces two signal-model-derived structural priors into representation learning: the spectro-temporal scale prior is encoded by learnable multi-resolution analysis, and the temporal-dependence prior is encoded by MCMM refinement before graph construction. Together, they guide the model to expose modulation evidence across complementary analysis scales and consolidate temporally dependent evidence before relational learning.

More specifically, the representation-learning part instantiates $P_{\mathrm{scale}}$ through learnable multi-resolution analysis and $P_{\mathrm{temporal}}$ through recurrent bidirectional temporal aggregation before adjacency estimation; CMCL regularizes the two views during training. The graph-learning part then forms self-loop, local temporal-chain, and top-*k* feature-similarity relations for graph-level classification.

### 3.2. Modulation-Prior-Enhanced Cross-Modal Encoding

This stage primarily instantiates the spectro-temporal scale prior $P_{\mathrm{scale}}$ in Equation (5). The first stage of MPCG-Net aims to construct complementary I/Q and spectro-temporal token representations from the received baseband signal. Under low-SNR conditions, a single representation may not reliably preserve sufficient modulation-discriminative information. Raw I/Q waveforms contain fine-grained amplitude and phase variations, but they are vulnerable to noise perturbations. Spectro-temporal representations reveal the energy distribution of the signal in the time-frequency domain, but a fixed-resolution time-frequency analysis may not simultaneously capture local transient changes and spectral structures. Therefore, instead of relying on a single input view, the proposed encoding stage adopts a dual-branch structure. The I/Q branch preserves local waveform evolution in the time domain, while the spectro-temporal branch extracts structural cues at different time-frequency resolutions through multi-resolution STFT analysis. These two views describe different aspects of the same modulation process and provide complementary evidence for subsequent cross-modal fusion and robust low-SNR recognition.

For the time-domain branch, the received I/Q segment $x\in R^{2\times L}$ is processed by a temporal encoder to obtain waveform tokens

(10) $$Z_{\mathrm{IQ}}=E_{\mathrm{IQ}}\left(x\right),\;Z_{\mathrm{IQ}}\in R^{T\times D},$$

where $E_{\mathrm{IQ}}\left(\cdot \right)$ denotes the I/Q encoder, *T* is the token length, and *D* is the token feature dimension. This branch mainly captures local amplitude and phase variations from the original complex baseband waveform.

For the spectro-temporal branch, the I/Q segment is first reconstructed as $r\left[n\right]=x_{I}\left[n\right]+jx_{Q}\left[n\right]$. Three STFT window lengths are used,

(11) $$w_{k}\in \left\{16,32,48\right\},\;k\in \left\{1,2,3\right\}.$$

For the *k*-th resolution, the standard discrete STFT is defined as

(12) $$X_{k}\left[m,q\right]=\sum_{n=0}^{L-1}r\left[n\right]g_{k}\left[n-mH\right]\exp\;\left(-j\frac{2\pi qn}{N_{\mathrm{FFT}}}\right),$$

where $g_{k}\left[\cdot \right]$ is the analysis window with length $w_{k}$, *H* is the hop size shared by all STFT branches, $N_{\mathrm{FFT}}$ is the common FFT length, *m* indexes the time frame, and *q* indexes the frequency bin. The corresponding log-magnitude spectro-temporal representation is computed as

(13) $$M_{k}\left[m,q\right]=\log\left(1+|X_{k}\left[m,q\right]|\right).$$

Because different window lengths may otherwise produce different numbers of raw time frames, MPCG-Net explicitly aligns the three STFT branches before weighted aggregation. In the implementation, all branches use the same FFT length $N_{\mathrm{FFT}}=128$ and hop size H=1, and the signal is center-padded with zeros during STFT computation. After magnitude and logarithmic compression, the first F=32 frequency bins are retained and the temporal dimension is cropped to the input length *L*. Therefore, each aligned spectrogram is represented as

(14) $${\tilde{M}}_{k}\in R^{T\times F},\;T=L,\;F=32.$$

No interpolation is applied after tokenization; the alignment is obtained by the common hop size, center padding, frequency-bin selection, and temporal cropping. Each aligned spectrogram is then normalized and projected into the common token space:

(15) $$Z_{\mathrm{ST},k}=E_{\mathrm{ST},k}\left({\tilde{M}}_{k}\right),\;Z_{\mathrm{ST},k}\in R^{T\times D}.$$

Different window lengths provide complementary time-frequency resolution characteristics. The short window emphasizes local temporal variations, the medium window balances temporal and spectral resolution, and the long window provides more stable frequency evidence under strong noise. To learn the relative contribution of each resolution, MPCG-Net uses softmax-normalized global learnable weights,

(16) $$\alpha_{k}=\frac{\exp(\theta_{k})}{\sum_{j=1}^{3}\exp\left(\theta_{j}\right)},\;\sum_{k=1}^{3}\alpha_{k}=1,$$

where $\theta_{k}$ denotes the learnable score of the *k*-th STFT branch. The final spectro-temporal token representation is

(17) $$Z_{\mathrm{ST}}=\sum_{k=1}^{3}\alpha_{k}Z_{\mathrm{ST},k},\;Z_{\mathrm{ST}}\in R^{T\times D}.$$

This design keeps the STFT front-end interpretable while allowing the network to learn a data-driven balance among different spectro-temporal resolutions.

**Figure 2:**
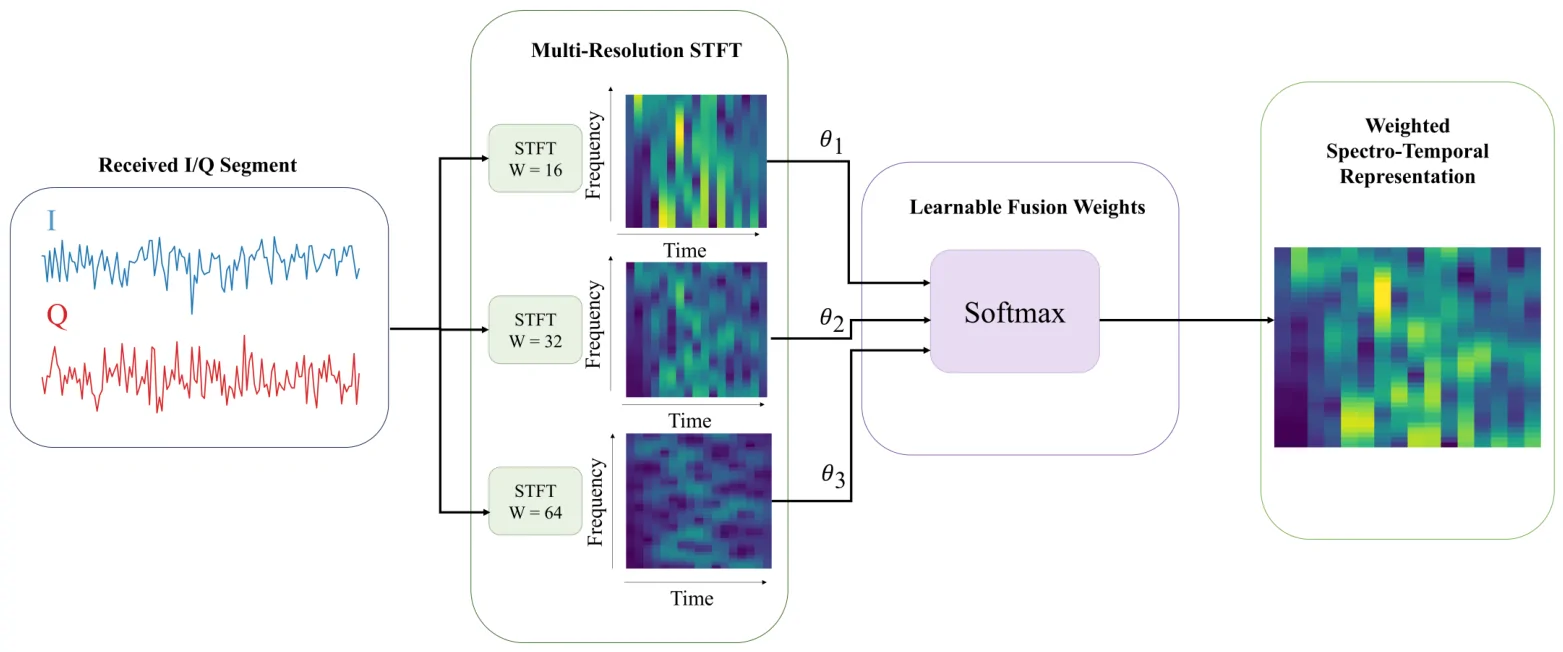
Learnable multi-resolution STFT encoding mechanism. The received I/Q segment is transformed into multiple spectro-temporal representations with different window lengths. Their token representations are projected into a shared feature space and fused through softmax-normalized global learnable weights to obtain the final spectro-temporal token sequence.

The learnable multi-resolution STFT encoding mechanism is illustrated in Figure 2.

During training, the two token sequences $Z_{\mathrm{IQ}}$ and $Z_{\mathrm{ST}}$ are further used for cross-modal consistency learning (CMCL). In this paper, CMCL denotes the high-level training objective that aligns the two modalities, while its concrete loss form is implemented as token-level distillation (TDL) between I/Q and spectro-temporal tokens. This auxiliary objective is not required during inference.

To enhance the interaction between the two modalities during training, MPCG-Net introduces an SNR-aware CMCL objective. The motivation is that the relative reliability of time-domain and spectro-temporal representations varies with the SNR level. Under strong noise, time-domain waveform patterns are more easily corrupted, and spectro-temporal representations may provide more stable structural cues. When the SNR increases, the original I/Q waveform becomes more reliable and can provide fine-grained modulation information. Accordingly, the CMCL loss is written as

(18) $$L_{\mathrm{cons}}=D\;\left(g_{s}\;\left(Z_{\mathrm{student}}\right),\;\mathrm{stopgrad}\;\left(g_{t}\;\left(Z_{\mathrm{teacher}}\right)\right)\right),$$

where D(·) denotes the token-level consistency distance, $g_{s}\left(\cdot \right)$ and $g_{t}\left(\cdot \right)$ are projection heads for the student and teacher representations, respectively, and stopgrad(·) prevents gradients from updating the teacher branch. In the implemented schedule, the spectro-temporal tokens guide the I/Q tokens in the designated low-SNR interval, the reverse direction is used in the designated high-SNR interval, and bidirectional consistency is adopted between them.

Finally, the I/Q and spectro-temporal token sequences are fused to form the cross-modal encoded representation:

(19) $$Z_{0}=\mathrm{Fuse}\left(Z_{\mathrm{IQ}},Z_{\mathrm{ST}}\right),$$

where Fuse(·) denotes the entrance fusion operation of the modulation context memory module: the two token sequences are concatenated along the feature dimension and projected by two 1×1 Conv1D layers, with batch normalization and GELU activation between them and dropout at the output. The resulting representation $Z_{0}\in R^{T\times D}$ contains both waveform-level and spectro-temporal modulation evidence for subsequent context refinement.

### 3.3. Modulation Context Memory Module

Guided by the temporal-dependence prior $P_{\mathrm{temporal}}$, MCMM aggregates surrounding signal evidence to refine each fused token before graph edges are estimated. Although the cross-modal encoding stage produces the fused representation $Z_{0}$, the resulting tokens may still contain fragmented or noise-dominated modulation evidence under low-SNR conditions. Directly constructing a graph from these tokens can make the subsequent adjacency estimation sensitive to local perturbations because unreliable token features may generate unstable temporal or similarity relations. As shown in Figure 1, MPCG-Net therefore inserts a modulation context memory module (MCMM) between cross-modal encoding and graph construction. The purpose of this module is not to introduce an additional input modality but to refine the fused token sequence by enhancing energy-related discriminative responses and preserving bidirectional temporal context before relational modeling. Here, the term ’memory’ refers to recurrent temporal memory inside the GRU and BiLSTM hidden states, rather than an external memory bank, memory queue, or separately maintained memory slots. In other words, MCMM stores and propagates modulation-related context along the token sequence through recurrent state transitions.

Let the batched fused representation obtained from the previous subsection be

(20) $$Z_{0}\in R^{B\times T\times D},$$

where *B* is the batch size, *T* is the token length, and *D* is the feature dimension of each fused token. For channel–temporal recalibration, $Z_{0}$ is first transposed as $U=Z_{0}^{⊤}\in R^{B\times D\times T}$. Since modulation differences are often reflected by amplitude- and energy-related variations, an energy-enhanced response is computed as

(21) $$E=U⊙U,\;E\in R^{B\times D\times T},$$

where ⊙ denotes element-wise multiplication. The energy response is then transposed along the temporal dimension and encoded by a gated recurrent unit (GRU) to model joint channel–temporal dependencies:

(22) $$H_{a}={\mathrm{GRU}}_{a}\left(E^{⊤}\right),\;H_{a}\in R^{B\times T\times 2D}.$$

A linear projection followed by a sigmoid activation produces the channel–temporal attention gate,

(23) $$G=\sigma {\left(W_{a}H_{a}+b_{a}\right)}^{⊤},\;G\in R^{B\times D\times T},$$

where $W_{a}$ and $b_{a}$ are learnable parameters and σ(·) is the sigmoid function. The recalibrated token response is obtained through residual gating:

(24) $$U_{a}=U⊙G+U.$$

This residual form allows the module to emphasize informative channel–temporal regions while preserving the original fused evidence, which is important when the attention gate is uncertain under strong noise.

After local recalibration, the token sequence is passed to a bidirectional temporal memory block. Specifically, the recalibrated sequence is transposed back to $U_{a}^{⊤}\in R^{B\times T\times D}$ and processed by a bidirectional LSTM:

(25) $${\vec{H}}_{t},{\overset{\leftarrow }{H}}_{t}=\mathrm{BiLSTM}\left(U_{a}^{⊤}\right),$$

(26) $$Z_{m}=\left[{\vec{H}}_{t};{\overset{\leftarrow }{H}}_{t}\right],\;Z_{m}\in R^{B\times T\times D_{m}},$$

where [·;·] denotes feature concatenation and $D_{m}=2H$ when the hidden size of each LSTM direction is *H*. The bidirectional recurrent states aggregate contextual evidence from both earlier and later positions, yielding a modulation-aware token sequence $Z_{m}$ for graph construction. Algorithm 1 summarizes MCMM, and the *i*-th token of $Z_{m}$ is used as the node feature $h_{i}$ in the next subsection.
**Algorithm 1** Modulation Context Memory Module**Input:** fused tokens $Z_{0}\in R^{B\times T\times D}$.
**Output:** refined tokens $Z_{m}\in R^{B\times T\times D_{m}}$.
**Channel–temporal recalibration:**
    Switch fused tokens to the channel-first layout: $U=Z_{0}^{⊤}$.
    Enhance energy-related modulation responses: E=U⊙U.
    Encode channel–temporal dependency: $H_{a}={\mathrm{GRU}}_{a}\left(E^{⊤}\right)$.
    Generate the recalibration gate: $G=\sigma {\left(W_{a}H_{a}+b_{a}\right)}^{⊤}$.
Preserve residual evidence: $U_{a}=U⊙G+U$.
**Bidirectional temporal memory:**
    Aggregate past and future context: $\left({\vec{H}}_{t},{\overset{\leftarrow }{H}}_{t}\right)=\mathrm{BiLSTM}\left(U_{a}^{⊤}\right)$.
    Form the refined token sequence: $Z_{m}=\left[{\vec{H}}_{t};{\overset{\leftarrow }{H}}_{t}\right]$.
**return** $Z_{m}$.


### 3.4. Dynamic Cross-Modal Graph Learning

**Figure 3:**
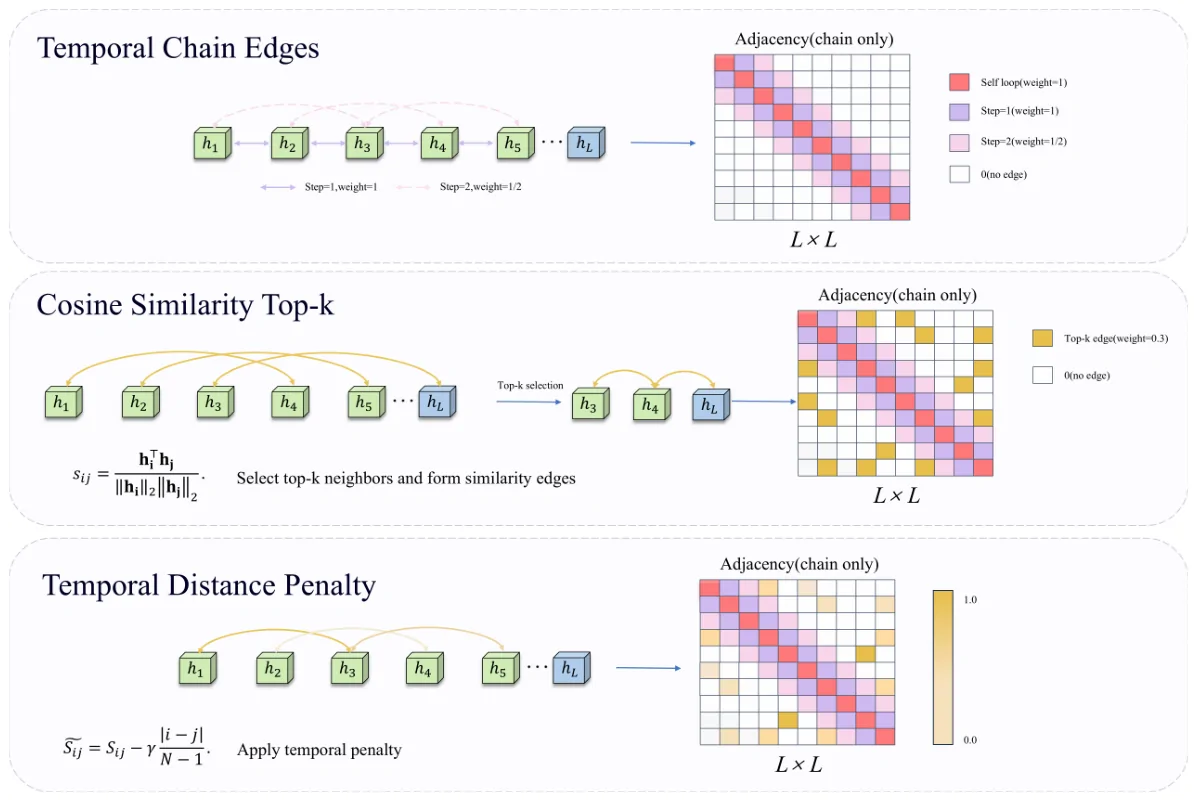
Dynamic temporal similarity graph construction and relational learning process. Refined tokens are converted into graph nodes, and self-loops, local temporal-chain edges, and top-*k* feature-similarity edges jointly define the adjacency matrix for DenseSAGEConv–DiffPool representation learning.

After the modulation context memory module, the refined token sequence $Z_{m}$ contains more stable local and contextual modulation evidence. However, these tokens are still arranged as a Euclidean sequence, whereas useful modulation cues under strong noise may appear not only between adjacent positions but also among distant tokens with similar spectral or temporal patterns. Treating the tokens as isolated sequence positions may therefore weaken non-local evidence aggregation. As illustrated in Figure 3, MPCG-Net converts $Z_{m}$ into a temporal similarity graph to jointly preserve local temporal continuity and adaptive feature-level relations.

Before graph construction, a feature pooling operation compresses the channel dimension of $Z_{m}$ and produces the graph node features

(27) $$X_{g}=\left\{h_{1},h_{2},\ldots ,h_{N}\right\},\;X_{g}\in R^{B\times N\times D_{g}},$$

where *N* is the number of graph nodes, $D_{g}$ is the graph-node feature dimension, and $h_{i}$ is the feature of the *i*-th token. MPCG-Net constructs an adjacency matrix $A\in R^{B\times N\times N}$ from three components:

(28) $$A=\mathrm{clip}\left(A_{\mathrm{self}}+A_{\mathrm{chain}}+A_{\mathrm{sim}},0,1\right).$$

The self-loop term $A_{\mathrm{self}}$ is an identity matrix, which preserves each token’s own evidence during graph propagation. The temporal-chain term connects nearby tokens within a window *r*:

(29) $$A_{\mathrm{chain}}\left(i,j\right)=\left\{\begin{array}{cc} 1/|i-j|, & 0<|i-j|\leq r,\\ 0, & \mathrm{otherwise}, \end{array}\right.$$

where the default chain window is r=2. This design keeps short-range temporal continuity and assigns stronger weights to closer temporal neighbors.

For non-local relation modeling, normalized token features are first used to compute cosine similarity,

(30) $$S_{ij}=\frac{h_{i}^{⊤}h_{j}}{∥h_{i}{∥}_{2}{∥h_{j}∥}_{2}}.$$

To avoid over-connecting distant noisy tokens only because of accidental similarity, a normalized temporal distance penalty is subtracted:

(31) $${\tilde{S}}_{ij}=S_{ij}-\gamma \frac{\left|i-j\right|}{N-1},$$

where γ is the temporal penalty coefficient. The diagonal entries are masked, and the top-*k* neighbors of each node are selected according to ${\tilde{S}}_{ij}$. The similarity adjacency is then defined as

(32) $$A_{\mathrm{sim}}\left(i,j\right)=\left\{\begin{array}{cc} \omega , & j\in N_{k}\left(i\right)\;\mathrm{or}\;i\in N_{k}\left(j\right),\\ 0, & \mathrm{otherwise}, \end{array}\right.$$

where $N_{k}\left(i\right)$ denotes the top-*k* feature-similar neighbors of node *i*, and ω is the similarity-edge weight. In the implementation, k=4, ω=0.3, and γ=0.15 are used by default.

Given $X_{g}$ and A, graph representation learning is performed by a hierarchical DenseSAGEConv–DiffPool backbone [46,48]. At the *l*-th pooling stage, an assignment matrix and node embeddings are computed as

(33) $$S^{\left(l\right)}={\mathrm{GNN}}_{\mathrm{pool}}^{\left(l\right)}\left(X^{\left(l\right)},A^{\left(l\right)}\right),\;Z^{\left(l\right)}={\mathrm{GNN}}_{\mathrm{embed}}^{\left(l\right)}\left(X^{\left(l\right)},A^{\left(l\right)}\right).$$

The graph is then coarsened by differentiable pooling:

(34) $$X^{\left(l+1\right)}={S^{\left(l\right)}}^{⊤}Z^{\left(l\right)},\;A^{\left(l+1\right)}={S^{\left(l\right)}}^{⊤}A^{\left(l\right)}S^{\left(l\right)}.$$

After two pooling stages, the final graph embeddings are further refined by DenseSAGEConv and averaged over nodes:

(35) $$g=\frac{1}{N^{\prime }}\sum_{i=1}^{N^{\prime }}x_{i}^{\prime }.$$

The graph-level representation g is passed through fully connected layers to obtain the final logits,

(36) $$\hat{y}=\mathrm{MLP}\left(g\right).$$

The overall graph construction and graph learning procedure is summarized in Algorithm 2.
**Algorithm 2** Temporal Similarity Graph Learning**Input:** refined tokens $Z_{m}\in R^{B\times T\times D_{m}}$.
**Output:** classification logits $\hat{y}$.
**Graph node preparation:**
    Compress the feature dimension and obtain node features $X_{g}\in R^{B\times N\times D_{g}}$.
**Graph structure construction:**
    Build self-loop edges $A_{\mathrm{self}}$ to preserve token identity.
    Build temporal-chain edges $A_{\mathrm{chain}}$ within window *r* to retain local continuity.
    Compute cosine similarity S between normalized node features.
    Apply temporal penalty ${\tilde{S}}_{ij}=S_{ij}-\gamma \left|i-j\right|/\left(N-1\right)$.
    Select top-*k* neighbors and form similarity edges $A_{\mathrm{sim}}$.
    Fuse edges: $A=\mathrm{clip}(A_{\mathrm{self}}+A_{\mathrm{chain}}+A_{\mathrm{sim}},0,1)$.
**Hierarchical graph learning:**
    Compute assignment and embedding matrices with DenseSAGEConv blocks.
    Apply DiffPool twice to obtain coarsened graph representations.
    Refine the final graph with DenseSAGEConv and average node embeddings.
**return** $\hat{y}=\mathrm{MLP}\left(g\right)$.


### 3.5. Classification and Training Objective

After hierarchical graph learning, the averaged graph-level representation g contains the final modulation-discriminative information of the received signal. A lightweight multilayer perceptron is used as the classifier to map this representation into the modulation label space. Specifically, g is first projected into a hidden representation,

(37) $$z_{c}=\mathrm{ReLU}\left(W_{1}g+b_{1}\right),$$

and then further transformed into the classification feature vector

(38) $$f_{c}=W_{2}z_{c}+b_{2}.$$

The final logits are obtained by a linear classification layer,

(39) $$\hat{y}=W_{3}f_{c}+b_{3},\;\hat{y}\in R^{C},$$

where *C* denotes the number of modulation classes. The predicted probability of the *c*-th modulation type is computed as

(40) $$p_{c}=\frac{\exp({\hat{y}}_{c})}{\sum_{j=1}^{C}\exp\left({\hat{y}}_{j}\right)}.$$

During training, MPCG-Net is optimized by combining the classification loss and the training-stage cross-modal consistency loss. The classification term is the cross-entropy loss defined in Equation (9), and the consistency term is defined in Equation (18). The total objective is

(41) $$L=L_{\mathrm{CE}}+\lambda_{\mathrm{cons}}L_{\mathrm{cons}},$$

where $\lambda_{\mathrm{cons}}$ controls the strength of cross-modal consistency regularization. Unless otherwise specified, $\lambda_{\mathrm{cons}}=0.3$ is used in all experiments. During inference, only the forward classification path is used; therefore, neither the SNR label nor the consistency loss is required.

## 4. Experimental Results and Analysis

### 4.1. Datasets

Experiments are conducted on three public AMR benchmark datasets. These datasets include RadioML2016.10A, RadioML2016.10B [58], and RML22 [59]. All datasets provide complex baseband I/Q samples with a length of 128, and each sample is associated with a modulation label and an SNR value. RadioML2016.10A and RadioML2016.10B are synthetic radio modulation benchmarks released by DeepSig, while RML22 is generated under more realistic wireless channel settings. In this paper, RadioML2016.10A and RadioML2016.10B are evaluated from −20 to 18 dB, and RML22 is evaluated from −20 to 20 dB. The low-SNR range used for focused comparison is −20 to 0 dB. All three datasets use discrete SNR levels with a spacing of 2 dB. Accordingly, the reported low-SNR accuracy is the arithmetic mean of the classification accuracies at the 11 SNR levels from −20 to 0 dB.

For each modulation class and SNR value, the samples are split into training, validation, and testing subsets with a ratio of 60%, 20%, and 20%, respectively. The split is performed within each class–SNR group to keep the modulation and SNR distributions consistent across the three subsets. The key characteristics of the datasets are summarized in Table 2.

RadioML2016.10A contains 11 modulation classes, including 8PSK, BPSK, CPFSK, GFSK, PAM4, QAM16, QAM64, QPSK, AM-DSB, AM-SSB, and WBFM. RadioML2016.10B removes AM-SSB and contains 10 classes with a larger number of samples per class–SNR pair. RML22 uses the same 11 modulation categories as RadioML2016.10A and extends the SNR range to 20 dB.

### 4.2. Network Configuration

Table 3 lists the main architectural settings used in the experiments. MPCG-Net is implemented as a dual-branch cross-modal graph learning network. The I/Q branch extracts waveform features directly from the received baseband signal, while the spectro-temporal branch constructs multi-resolution STFT representations and projects them into the same token space. The fused tokens are refined by the modulation context memory module and then converted into a temporal similarity graph for graph-level classification. The number of output classes is set according to the target dataset; therefore, only the last classification layer changes between the 11-class datasets and RadioML2016.10B. All other architectural settings are kept the same unless explicitly specified in an ablation variant.

### 4.3. Experimental Setup

All experiments are implemented in PyTorch and trained on NVIDIA RTX 3090 GPUs. AdamW is used as the optimizer with an initial learning rate of $1\times 10^{-3}$. A ReduceLROnPlateau scheduler is used. The maximum number of training epochs is set to 100. The batch size is 256 for training and 400 for validation and testing. The training objective follows Equation (41) with $\lambda_{\mathrm{cons}}=0.3$.

### 4.4. Comparison with Baseline Models

To evaluate the recognition performance of MPCG-Net, five representative AMR baselines are selected for comparison. These baselines have been introduced in Section 1; for clarity, their modeling characteristics are briefly summarized here. MCLDNN [33] learns spatiotemporal multi-channel representations from I/Q signals through convolutional and recurrent modeling. AMCNet [34] improves AMR through frequency-domain denoising and multi-scale feature extraction. AvgNet [51] represents radio signals with adaptive visibility graphs and performs graph-based classification. FEA-T [35] uses frame-wise embedding and Transformer modeling to capture global signal correlations. IQFormer [43] is a Transformer-based multimodal AMR model that combines I/Q and time-frequency representations.

For a fair comparison, all baseline results reported in this paper are obtained by re-training the corresponding models in the same experimental environment rather than directly copying results from the original papers. The baselines and MPCG-Net use the same training, validation, and testing splits described in Section 4, and they are trained with the same optimizer, initial learning rate, learning-rate scheduler, maximum number of epochs, batch size, and evaluation protocol listed in Table 4. The network architectures and main hyperparameters of the baselines follow their original papers or available implementations as closely as possible. Only dataset-dependent components, such as the final classification layer and necessary input-format adapters, are adjusted to match the number of modulation classes and the input representation used by each baseline. For I/Q-only baselines, no additional spectro-temporal input branch is introduced; IQFormer keeps its multimodal preprocessing setting.

Table 5 reports the overall test accuracy and the average accuracy in the low-SNR range from −20 to 0 dB. The table also reports parameter count, neural-layer multiply–accumulate operations (MACs), and measured end-to-end CPU latency to compare recognition performance with computational cost.

MPCG-Net achieves the best overall and low-SNR accuracy on all three datasets. Compared with the strongest baseline on each dataset, namely IQFormer, its low-SNR average accuracy improves by 6.90, 6.45, and 1.66 percentage points on RadioML2016.10A, RadioML2016.10B, and RML22, respectively. These results demonstrate consistent low-SNR improvements across all three datasets.

CPU latency is measured on an Intel Xeon Silver 4210. For every model, inference is warmed up for 10 runs and then repeated 50 times under the same single-thread setting; Table 5 reports the median single-sample end-to-end latency. The consistent input, execution setting, and warm-up procedure reduce measurement bias and provide a uniform comparison across models.

Although MPCG-Net has only 20.13 M reported neural-layer MACs, its measured latency is higher than that of the other models. This difference arises because the reported MACs do not include the three-resolution STFT front-end, dynamic top-*k* similarity and adjacency construction, or graph pooling operations. These stages also involve memory movement and small, dynamically organized operations whose CPU execution efficiency is not reflected by the MAC count. Therefore, neural-layer MACs should not be interpreted as a direct proxy for end-to-end wall-clock latency. Nevertheless, MPCG-Net completes single-sample end-to-end inference in a median of 32.66 ms on a single CPU thread, remaining within a low-latency inference regime while providing the strongest low-SNR recognition accuracy.

**Figure 4:**
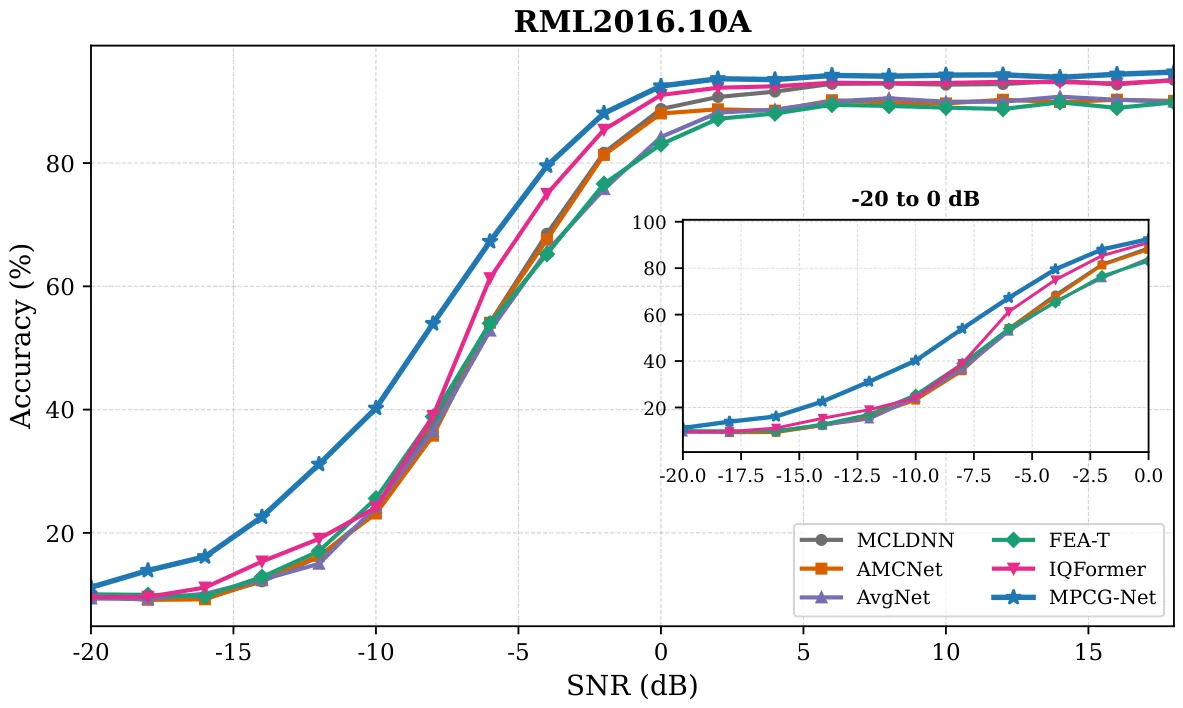
Accuracy–SNR comparison on RadioML2016.10A. The inset enlarges the low-SNR range from −20 to 0 dB.

**Figure 5:**
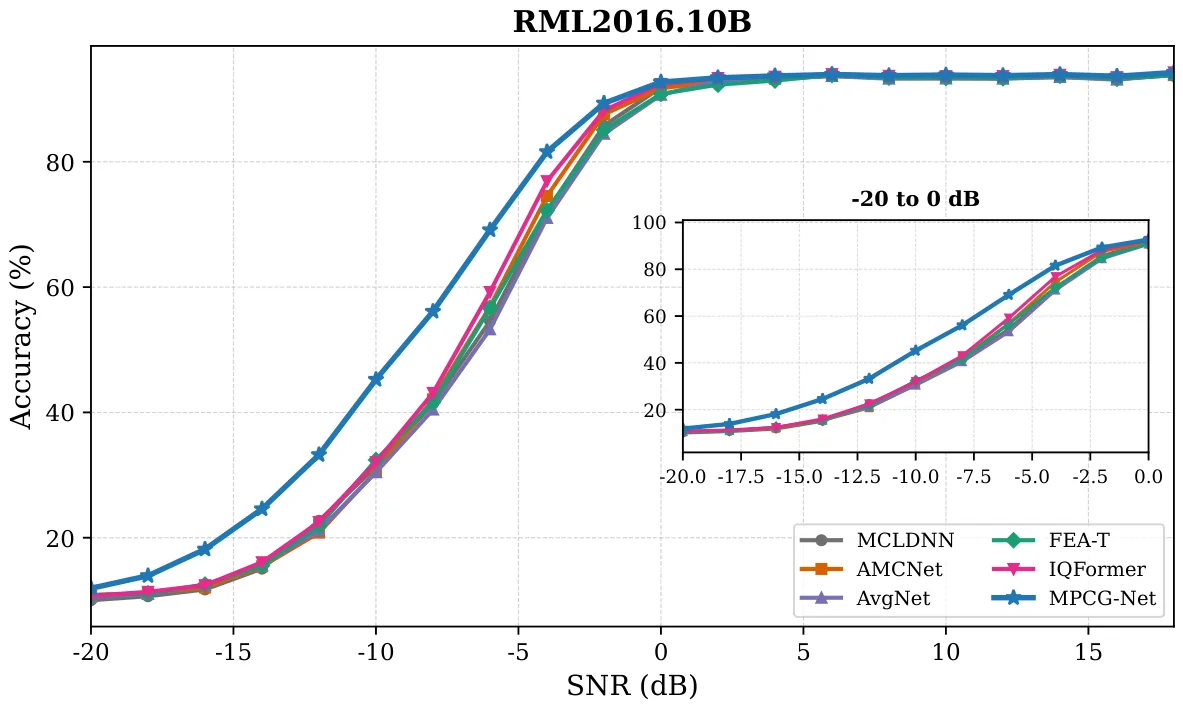
Accuracy–SNR comparison on RadioML2016.10B. The inset enlarges the low-SNR range from −20 to 0 dB.

**Figure 6:**
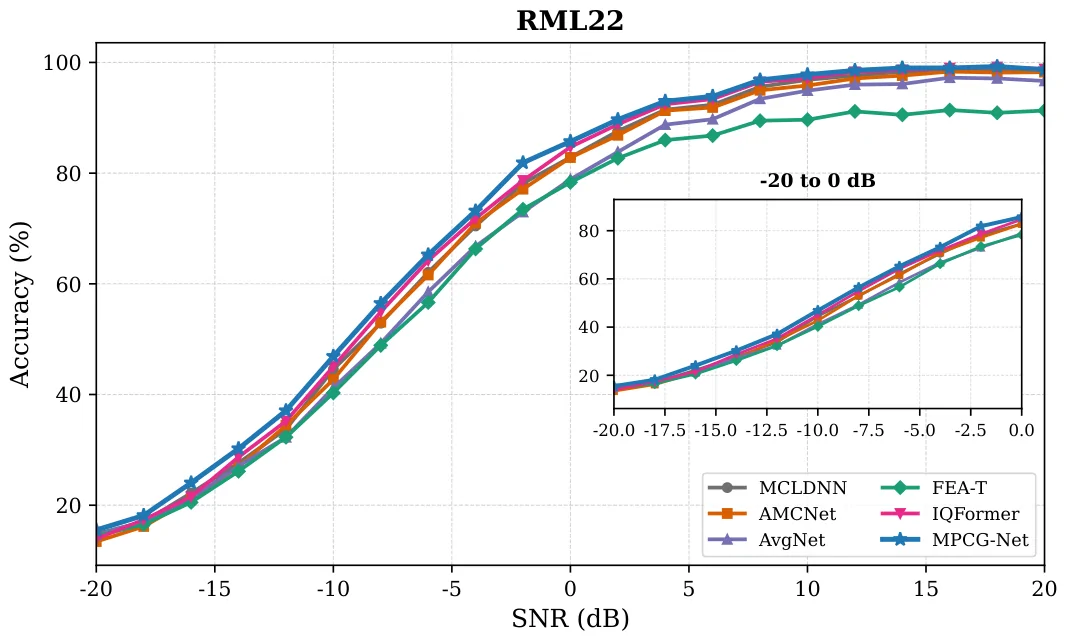
Accuracy–SNR comparison on RML22. The inset enlarges the low-SNR range from −20 to 0 dB.

Figure 4, Figure 5 and Figure 6 further show the accuracy-SNR curves on the three datasets. The enlarged regions highlight the −20 to 0 dB interval. MPCG-Net consistently produces higher recognition accuracy in this low-SNR range while maintaining competitive or better performance at medium and high SNR levels.

Figure 4, Figure 5 and Figure 6 show that the proposed MPCG-Net maintains a consistent advantage over the baseline models in the low-SNR range. This indicates that the improvement is not caused by isolated SNR points but by more stable feature learning under severe noise corruption. As the SNR increases, the performance gap between different models gradually becomes smaller because modulation-discriminative waveform and spectral patterns become easier to extract. On RML22, the gain is relatively smaller than that on RadioML2016.10A and RadioML2016.10B, which may be attributed to the more realistic signal generation setting and the strong multimodal modeling ability of IQFormer. Nevertheless, MPCG-Net still achieves the best low-SNR average accuracy, demonstrating the robustness of the proposed cross-modal graph learning framework.

### 4.5. Robustness Evaluation with Measured RF Interference

To evaluate robustness to structured interference beyond the original benchmark, a hybrid test with measured RF interference is conducted on RadioML2016.10A. Desired signals are drawn from its test set, while the added interference comprises four waveform types from the RF Challenge dataset [60]: EMISignal1, CommSignal2, CommSignal3, and CommSignal5G1. EMISignal1 is electromagnetic interference caused by unintentional radiation from a man-made source; CommSignal2 and CommSignal3 are two distinct digital communication signals emitted by commercially available wireless devices; and CommSignal5G1 is a 5G-compliant waveform. The first three were recorded over the air, whereas CommSignal5G1 was generated and recorded in a controlled wired laboratory environment.

For each RadioML sample, a contiguous 128-point interference segment is randomly cropped, phase-rotated, and scaled to a target signal-to-interference ratio (SIR) of −5, 0, 5, or 10 dB. Corresponding samples across SNR levels use the same interference frame, crop, and phase, and all models share the resulting mixtures. The original checkpoints are evaluated without retraining over the −20 to 0 dB SNR range.

As shown in Table 6, MPCG-Net achieves the highest average accuracy under every interference type and outperforms the strongest baseline, IQFormer, by 5.88 percentage points overall. Its improvement over IQFormer ranges from 3.80 to 7.34 percentage points across the four interference types. These consistent gains indicate that the improvement is not driven by a single interference category and demonstrate the robustness of MPCG-Net to measured RF interference waveforms that were not used during training, without requiring retraining or fine-tuning.

### 4.6. Component Ablation Study

To further verify the contribution of the main components in MPCG-Net, a component ablation study is conducted on RadioML2016.10A, RadioML2016.10B, and RML22. The ablated components are selected according to the architecture described in Section 3. Specifically, w/o learnable MRA replaces the learnable multi-resolution STFT weighting in Section 3.2 with fixed equal weights, so that the three STFT resolutions contribute equally. w/o CMCL removes the cross-modal consistency learning objective in Equation (18). Since CMCL is implemented by the token-level distillation loss between I/Q and spectro-temporal tokens, this variant disables the TDL implementation during training. w/o MCMM removes the modulation context memory module described in Section 3.3 and directly feeds the fused cross-modal tokens into the graph-based relational learning stage. The full model keeps all three components and is treated as the complete MPCG-Net configuration.

Table 7 shows that the full MPCG-Net achieves the best overall and low-SNR accuracy on all three datasets, confirming that the proposed components are complementary rather than isolated improvements. Removing the learnable multi-resolution STFT weighting leads to the largest performance degradation on RadioML2016.10A and RadioML2016.10B, which indicates that adaptively combining different time-frequency resolutions is important for the two RadioML2016 benchmarks. This observation is consistent with the motivation in Section 3.2: a single fixed time-frequency resolution may not simultaneously preserve local transient changes and stable spectral structures under low-SNR conditions.

The ablation of CMCL reduces recognition accuracy, showing that its auxiliary regularization promotes useful information exchange between the I/Q and spectro-temporal branches. Removing MCMM also decreases accuracy, indicating that temporal-dependence-guided refinement consolidates useful modulation evidence before graph construction. Overall, these results support the two connected parts of MPCG-Net: modulation-prior-enhanced cross-modal representation learning improves node features, while graph-based relational learning captures adaptive local and non-local relations among the refined tokens.

To separate the effects of input modality, spectro-temporal scale modeling, auxiliary consistency training, contextual refinement, and graph learning, we further conduct a controlled decomposition on RadioML2016.10A. Table 8 starts from two single-branch models and then evaluates a fixed-resolution multimodal model, its learnable MRA counterpart, the separate additions of CMCL and MCMM, the complete representation-learning model without the graph stage, and the full MPCG-Net. The CMCL and MCMM rows are parallel controlled variants rather than a strictly sequential cumulative path. All configurations use the same data split, maximum number of epochs, early-stopping criterion, and checkpoint-selection rule.

The fixed-resolution multimodal model improves overall and low-SNR accuracy by 3.42 and 2.46 percentage points, respectively, over the stronger single branch, confirming that I/Q and spectro-temporal inputs provide complementary evidence. Replacing fixed STFT-32 with learnable MRA, at the same reported parameter count, further improves the two metrics by 4.09 and 7.24 percentage points. This is the largest controlled increment and identifies learnable multi-resolution representation as the primary performance source in the present framework. Relative to I/Q + learnable MRA, adding CMCL provides modest gains of 0.11 and 0.16 percentage points, whereas adding MCMM yields gains of 1.32 and 0.90 percentage points. CMCL is therefore interpreted as an auxiliary regularizer, while MCMM provides an additional contribution by refining temporally dependent evidence before relational modeling.

The full model without graph learning reaches 66.17% overall accuracy and 44.87% low-SNR accuracy. Adding the complete graph-learning stage increases these values by 2.02 and 2.09 percentage points. Because this comparison also introduces the graph backbone, hierarchical pooling, graph-level classification, and additional parameters, it measures the aggregate contribution of the graph stage rather than isolating the proposed adjacency topology. The topology-specific contribution is examined under matched graph backbones and parameter counts in Section 4.8.

### 4.7. Multi-Resolution STFT Ablation

The component ablation in Table 7 shows that replacing the learnable multi-resolution aggregation (MRA) with equal weights causes a clear performance drop. To further analyze this front-end design, this subsection evaluates different MRA settings on the three datasets. The full MPCG-Net uses learnable weights to combine three STFT resolutions. The equal-weight variant fixes the three resolution weights to 1/3. The single-resolution variants keep only one STFT branch, where the short, medium, and long windows correspond to window lengths of 16, 32, and 48, respectively.

As shown in Table 9, the learnable MRA setting achieves the best overall and low-SNR accuracy on all three datasets. The equal-weight variant is better than relying on a single fixed resolution in some cases, but it still underperforms the learnable setting. This indicates that simply combining multiple STFT resolutions is not sufficient; the model also needs to adjust their relative contributions during training. Among the single-resolution variants, the medium window is generally stronger than the short and long windows, suggesting that the 32-point window provides a useful balance between local temporal resolution and frequency stability. Nevertheless, the single-window settings consistently lag behind the learnable multi-resolution design, which confirms that modulation evidence is distributed across different time-frequency scales.

**Figure 7:**
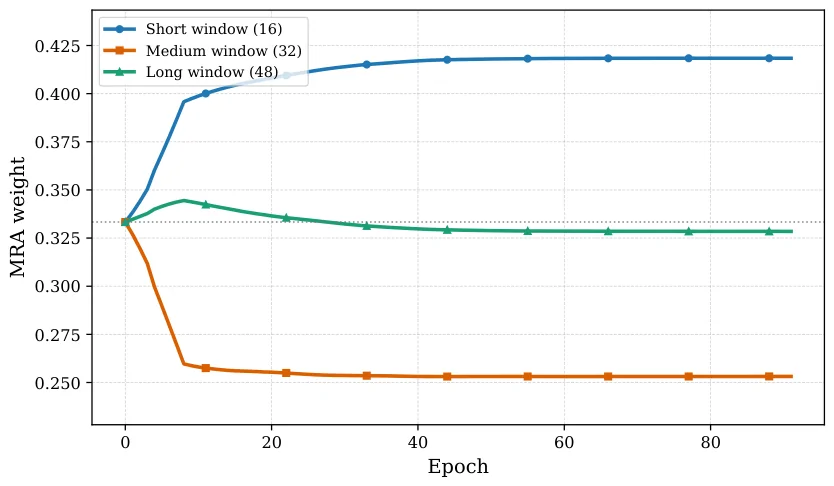
Evolution of learnable MRA weights on RadioML2016.10A. The short, medium, and long windows correspond to STFT window lengths of 16, 32, and 48, respectively.

To verify that the MRA weights are actually optimized rather than remaining fixed, Figure 7 plots the training evolution of the three resolution weights on RadioML2016.10A. The curves start from the uniform value 1/3 and gradually diverge as training proceeds. The short-window weight increases, while the medium- and long-window weights decrease to different degrees. This trend indicates that the front-end learns a data-driven preference among the three STFT resolutions instead of using a static handcrafted combination.

### 4.8. Multi-Seed Graph Construction and CMCL Coefficient Ablation

After the multi-resolution front-end and the modulation context memory module, MPCG-Net constructs a temporal similarity graph for graph-based relational learning. To distinguish the aggregate contribution of the graph stage from the contribution of the proposed adjacency topology, we repeat the graph ablation on RadioML2016.10A using five random seeds. The without-graph variant removes graph propagation and hierarchical pooling. All graph variants retain the same feature encoder, DenseSAGEConv–DiffPool backbone, classifier, and parameter count while changing only the available relations: the identity graph retains self-loops only; chain only adds local temporal-chain edges; similarity only adds temporally penalized feature-similarity edges; no temporal penalty retains both edge types but removes the distance penalty; and the full graph includes all components. The CMCL coefficient study subsequently keeps the full graph fixed and changes only the weight $\lambda_{\mathrm{cons}}$ of the auxiliary consistency term.

Table 10 shows that the complete graph stage improves the mean overall and low-SNR accuracies by 1.80 and 1.92 percentage points, respectively, over the without-graph model. However, this comparison includes the combined effects of the graph backbone, DiffPool-based hierarchical aggregation, graph-level classification, and the increase in model capacity. The identity graph alone recovers 1.64 percentage points on both metrics relative to the without-graph model, indicating that most of the graph-stage gain is associated with these shared graph-processing components rather than with the proposed relation topology alone.

Under the matched 0.335 M-parameter graph backbone, the full temporal similarity graph improves overall and low-SNR accuracy by 0.16 and 0.28 percentage points over the identity graph. It also exceeds the chain-only and similarity-only variants by 0.10–0.13 percentage points overall and 0.22 percentage points at low SNR, showing a small complementary trend between local temporal continuity and non-local feature similarity. Adding the temporal-distance penalty improves the corresponding means by 0.04 and 0.17 percentage points over the no-penalty variant. Therefore, the graph-learning stage provides a clear aggregate benefit, whereas the additional contribution of the specific mixed topology is positive but limited, and the temporal penalty provides only a modest mean improvement.

**Figure 8:**
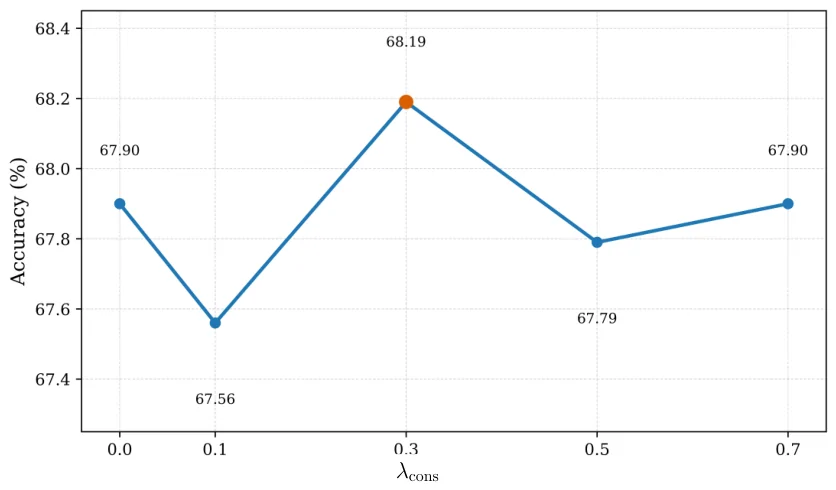
Effect of the CMCL coefficient $\lambda_{\mathrm{cons}}$ on RadioML2016.10A. The value beside each point denotes the corresponding test accuracy.

The coefficient $\lambda_{\mathrm{cons}}$ controls the contribution of the CMCL loss in Equation (18). Figure 8 reports the recognition accuracy under different $\lambda_{\mathrm{cons}}$ values. When $\lambda_{\mathrm{cons}}$ is too small, the two modalities are weakly constrained and the complementary relationship between I/Q and spectro-temporal tokens is not sufficiently regularized. When $\lambda_{\mathrm{cons}}$ becomes too large, the consistency objective may over-constrain the two branches and reduce their capacity to learn modality-specific representations. The best result is obtained at $\lambda_{\mathrm{cons}}=0.3$, which is therefore used as the default setting in the main experiments.

### 4.9. Visualization Analysis

Quantitative results show the recognition advantage of MPCG-Net, while visualization provides a more intuitive view of its class-level behavior and learned representation structure. To keep the presentation concise while covering all three benchmarks, this subsection reports confusion matrices and t-SNE scatter plots at representative low-SNR operating points of 0, −4, and −10 dB. For each dataset, the visualizations are generated from the selected model checkpoints used in the corresponding quantitative evaluation.

**Figure 9:**
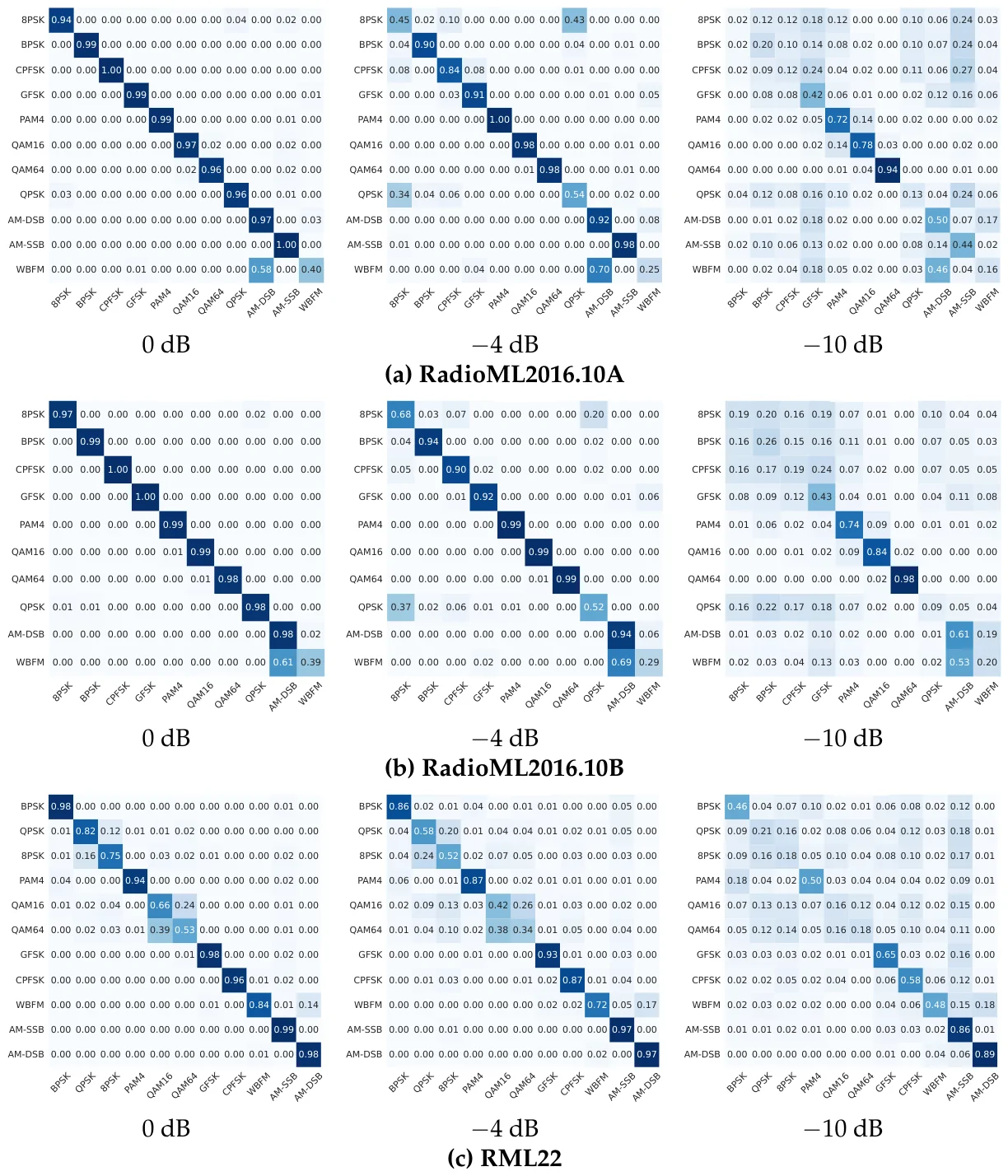
Confusion matrices at 0, −4, and −10 dB on the three benchmark datasets. In each row, the left, middle, and right panels correspond to 0, −4, and −10 dB, respectively.

Figure 9 shows that the diagonal structure becomes progressively weaker as the SNR decreases from 0 to −10 dB, but recognizable class-wise discrimination is still preserved across the three benchmarks. The degradation pattern is dataset-dependent: RadioML2016.10A and RadioML2016.10B mainly exhibit increased confusion among modulation formats with similar local structures, whereas RML22 shows a broader spread caused by its more realistic and diverse signal conditions.

**Figure 10:**
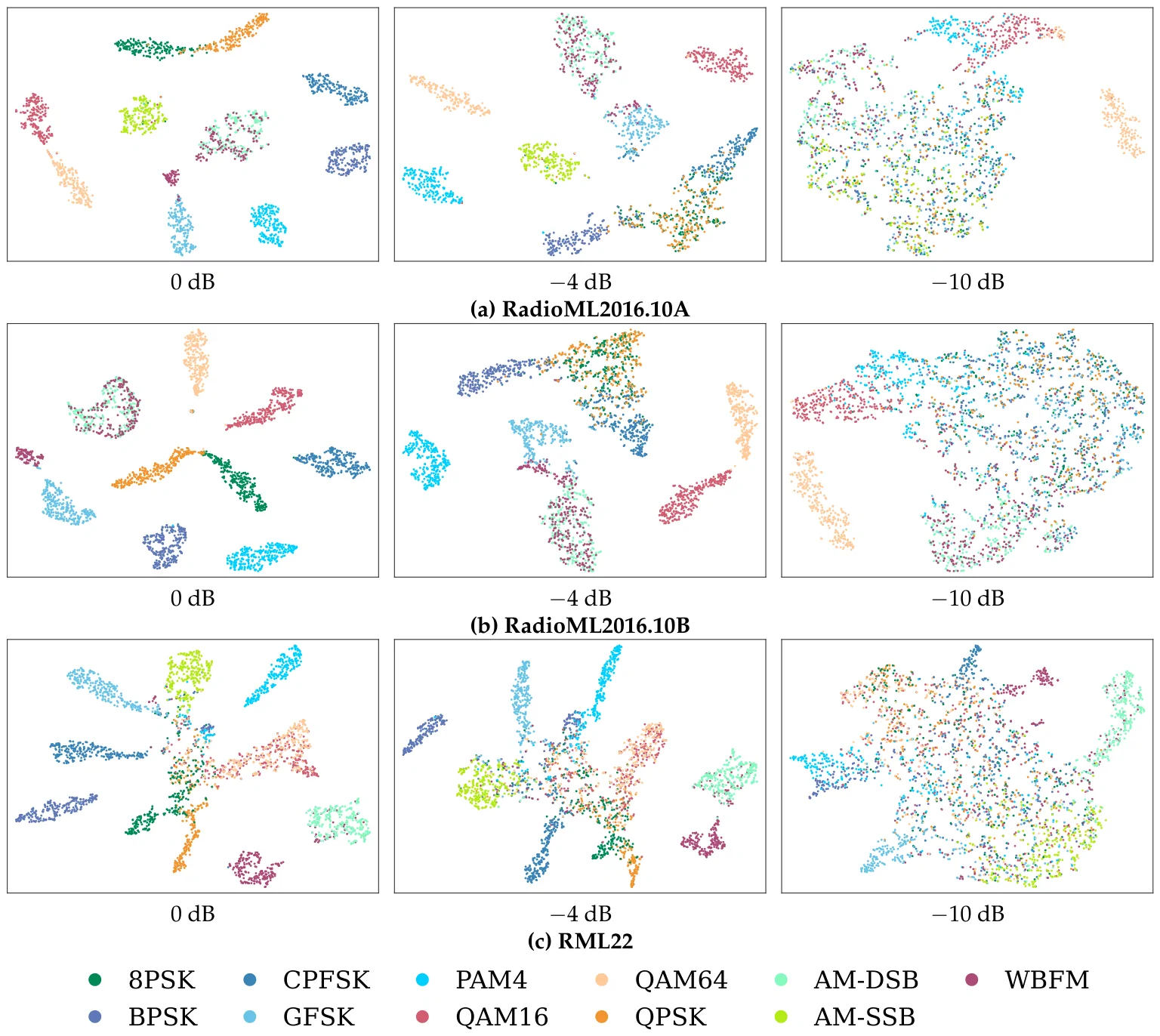
t-SNE visualizations at 0, −4, and −10 dB on the three benchmark datasets. In each row, the left, middle, and right panels correspond to 0, −4, and −10 dB, respectively. The shared color legend applies to all three datasets.

Figure 10 further shows that the learned representation manifold becomes less compact as the SNR decreases, but clear clustering trends remain visible even at −10 dB. The cross-dataset consistency of this trend supports the effectiveness of the two-part design, in which modulation-prior-enhanced cross-modal representation learning first forms robust node features and graph-based relational learning then organizes their local and non-local dependencies.

## 5. Conclusions

This paper presents MPCG-Net, a modulation-prior-enhanced cross-modal graph learning framework for robust automatic modulation recognition under low-SNR conditions. Motivated by the reduced reliability of parameter-dependent evidence under strong noise, we analyze common modulation signal models and summarize two structural priors: the spectro-temporal scale prior and the temporal-dependence prior. MPCG-Net follows a two-part processing flow. In the representation-learning part, a learnable multi-resolution STFT encoder exposes modulation evidence at complementary analysis scales, cross-modal consistency learning regularizes the interaction between I/Q and spectro-temporal tokens, and the modulation context memory module consolidates temporally dependent evidence before graph construction. In the graph-learning part, a temporal similarity graph preserves short-range temporal continuity while introducing data-dependent non-local relations for hierarchical graph-level classification.

The main findings of this study are summarized as follows.

1. MPCG-Net provides a unified two-part framework in which modulation-domain knowledge first guides cross-modal representation formation and graph learning subsequently organizes relations among the refined tokens. This two-stage pipeline connects complementary time-frequency evidence with hierarchical dependencies among signal segments.
2. The learnable multi-resolution STFT front-end is the most influential component in the proposed framework. Compared with fixed-resolution or equal-weight variants, the learnable aggregation strategy provides stronger low-SNR performance, empirically supporting the spectro-temporal scale prior that modulation evidence is distributed across complementary time–frequency resolutions.
3. Cross-modal consistency learning, the modulation context memory module, and graph learning provide supplementary gains beyond the dominant contribution of learnable multi-resolution representation. In the five-seed study, the complete graph stage improves mean overall and low-SNR accuracy by 1.80 and 1.92 percentage points over the without-graph model; this aggregate gain includes the graph backbone, hierarchical pooling, graph-level classification, and additional capacity. Under a matched graph backbone and parameter count, the full temporal similarity topology provides smaller gains of 0.16 and 0.28 percentage points over the identity graph. Thus, local temporal and non-local similarity relations show a complementary trend, but their topology-specific contribution is incremental rather than the primary source of the reported performance improvement.
4. Across RadioML2016.10A, RadioML2016.10B, and RML22, MPCG-Net reaches overall accuracies of 68.19%, 69.01%, and 71.44%, and low-SNR accuracies of 46.96%, 48.73%, and 48.59%, respectively. Relative to the strongest baseline on each dataset, the corresponding low-SNR gains are 6.90, 6.45, and 1.66 percentage points, respectively. MPCG-Net also achieves the best aggregate accuracy across all four measured RF interference categories without retraining.

Although the proposed framework shows consistent improvements on three public AMR benchmarks, several issues remain worthy of further study. First, the current multi-resolution front-end is based on predefined STFT window lengths, and more general learnable or signal-adaptive time-frequency transforms may further improve robustness. Second, the present evaluation focuses on closed-set modulation recognition; open-set recognition, unknown signal rejection, and cross-channel generalization are important for practical non-cooperative scenarios. Third, deployment on real radio hardware requires additional analysis of computational efficiency, channel mismatch, and online inference stability. Future work will investigate these directions to further improve the practical applicability of low-SNR AMR systems.

## Figures and Tables

**Table 1:** Comparison of representative multimodal and graph-based AMR methods.

Method	Input Representation	Fusion Strategy	Auxiliary Supervision	Graph Construction Strategy	Training Objective
AbFTNet [41]	I/Q + FRFT	Pre-alignment and cross-modal aggregation	CPC contrast	None	$L_{\mathrm{CE}}+\alpha L_{\mathrm{CPC}}$
IQFormer [43]	I/Q + STFT	Concatenation, 1×1 Conv, and BiLSTM	None	None	$L_{\mathrm{CE}}$
AvgNet [51]	I/Q	Late fusion of I/Q graph streams	None	Adaptive visibility graph + DiffPool	$L_{\mathrm{CE}}$
CTGNet [52]	Windowed I/Q	Late fusion of I/Q graph streams	None	Transformer adjacency + GraphSAGE–DMoNPool	$L_{\mathrm{CE}}$
STF-GCN [53]	I/Q + spatial augmentation + STFT	CNN–BiLSTM feature embedding	None	Adaptive correlation + GCN–PoolGAT	$L_{\mathrm{CE}}$

**Table 2:** Summary of the datasets used in the experiments.

Dataset	Source	Channel Setting	Classes	SNR Range	Samples
RML2016.10A [58]	Synthetic	Simulated	11	−20–18 dB	220,000
RML2016.10B [58]	Synthetic	Simulated	10	−20–18 dB	1,200,000
RML22 [59]	Realistic simulation	Realistic	11	−20–20 dB	462,000

**Table 3:** Main network configuration of MPCG-Net.

Component	Setting
Input	I/Q segment, 2×128
I/Q encoder	Grouped Conv1D, 2→16, kernel size 5
Spectro-temporal encoder	STFT windows {16,32,48}, FFT length 128, 32 frequency bins
STFT projection	Conv2D, 1→16, kernel size (32,1)
Modulation context memory module	Cross-modal 1×1 Conv fusion, GRU-based channel–temporal recalibration, and two-layer BiLSTM refinement; 32 input channels and 64 output channels
Graph construction	Self-loop + temporal chain (r=2) + top-*k* similarity (k=4, ω=0.3, γ=0.15)
Graph learning	DenseSAGEConv–DiffPool backbone, hidden size 64, two pooling stages
Classifier	MLP, 256→128→64→C
Trainable parameters	334,624 for 11 classes; 334,559 for 10 classes

**Table 4:** Experimental environment and training settings.

Item	Setting
GPU	NVIDIA GeForce RTX 3090 GPUs
Framework	PyTorch 1.10.1 + CUDA 11.1; PyTorch Geometric 2.4.0
Optimizer	AdamW
Initial learning rate	$1\times 10^{-3}$
Learning-rate scheduler	ReduceLROnPlateau
Training epochs	Maximum of 100 epochs
Batch size	256 for training; 400 for validation and testing
Dataset split	60% training, 20% validation, and 20% testing within each class–SNR group
Training objective	Equation (41), with $\lambda_{\mathrm{cons}}=0.3$

**Table 5:** Comparison with representative AMR baseline models on three datasets. Overall and low-SNR accuracies are reported in percentages; latency is the median single-sample end-to-end CPU latency.

Model	Params (M)	MACs (M)	Latency (ms)	RadioML2016.10A		RadioML2016.10B		RML22	
				**Overall**	−20**–**0 **dB**	**Overall**	−20**–**0 **dB**	**Overall**	−20**–**0 **dB**
MCLDNN [33]	0.369	41.88	10.68	62.23	37.40	64.53	40.66	69.65	46.04
AMCNet [34]	0.466	29.82	4.39	60.70	36.97	64.71	41.25	69.08	45.49
AvgNet [51]	0.456	36.78	18.90	60.19	35.95	64.08	40.10	67.32	43.63
FEA-T [35]	0.170	6.65	6.83	60.17	36.64	64.54	40.98	64.95	43.08
IQFormer [43]	0.355	45.12 *	27.50	63.85	40.06	65.44	42.28	70.40	46.93
MPCG-Net	0.335	20.13 *	32.66	**68.19**	**46.96**	**69.01**	**48.73**	**71.44**	**48.59**

* The reported neural-layer MACs exclude STFT preprocessing and dynamic graph operations. Bold values indicate the best result in each column; the same convention is used in the subsequent result tables.

**Table 6:** Low-SNR accuracy (%) under four types of measured RF interference on RadioML2016.10A. Each interference-specific result is the mean over the −20 to 0 dB SNR range and four target SIRs (−5, 0, 5, and 10 dB). Results are reported as mean ± standard deviation over five independently sampled interference mixtures. Overall denotes the equal-weight average across the four interference types.

Model	EMISignal1	CommSignal2	CommSignal3	CommSignal5G1	Overall
MCLDNN [33]	25.15±0.34	28.37±0.13	27.52±0.07	25.38±0.15	26.61±0.03
AMCNet [34]	25.72±0.25	29.97±0.09	28.50±0.13	26.10±0.24	27.57±0.05
AvgNet [51]	24.44±0.15	28.18±0.15	28.14±0.06	25.44±0.19	26.55±0.05
FEA-T [35]	23.99±0.28	26.79±0.06	25.36±0.11	24.41±0.13	25.14±0.06
IQFormer [43]	26.49±0.26	31.70±0.10	30.18±0.07	27.10±0.19	28.87±0.06
**MPCG-Net**	33.83±0.23	35.50±0.09	35.65±0.14	34.02±0.19	34.75±0.09

**Table 7:** Component ablation results on three datasets. Overall and low-SNR accuracies are reported in percentages, and the parameter count is reported in millions.

Variant	Params (M)	RadioML2016.10A		RadioML2016.10B		RML22	
		**Overall**	−20**–**0 **dB**	**Overall**	−20**–**0 **dB**	**Overall**	−20**–**0 **dB**
Full MPCG-Net	0.335	**68.19**	**46.96**	**69.01**	**48.73**	**71.44**	**48.59**
w/o learnable MRA	0.335	63.58	39.09	65.40	42.17	71.05	47.74
w/o CMCL	0.335	67.97	46.57	68.71	48.12	70.82	47.39
w/o MCMM	0.274	67.26	46.11	68.05	47.51	70.34	47.34

**Table 8:** Controlled decomposition of MPCG-Net on RadioML2016.10A. Overall and low-SNR accuracies are reported in percentages.

Configuration	Params	Overall	−20–0 dB
I/Q branch only	50,127	56.83	32.89
STFT-32 branch only	50,652	57.26	34.21
I/Q + fixed STFT-32	51,264	60.68	36.67
I/Q + learnable MRA	51,264	64.77	43.91
I/Q + MRA + CMCL	51,808	64.88	44.07
I/Q + MRA + MCMM	112,032	66.09	44.81
Full without graph	112,576	66.17	44.87
Full MPCG-Net	334,624	**68.19**	**46.96**

**Table 9:** Ablation results of the multi-resolution STFT front-end. Overall and low-SNR accuracies are reported in percentages.

Variant	RadioML2016.10A		RadioML2016.10B		RML22	
	**Overall**	−20**–**0 **dB**	**Overall**	−20**–**0 **dB**	**Overall**	−20**–**0 **dB**
Learnable MRA	**68.19**	**46.96**	**69.01**	**48.73**	**71.44**	**48.59**
Equal weights	63.58	39.09	65.40	42.17	71.05	47.74
Single short window (16)	63.56	39.11	65.27	41.96	70.74	47.19
Single medium window (32)	63.67	39.32	65.43	42.24	71.27	48.02
Single long window (48)	63.36	38.96	65.18	41.83	70.53	47.12

**Table 10:** Five-seed graph ablation on RadioML2016.10A. Results are reported as mean ± standard deviation in percentages.

Variant	Params (M)	Overall	−20–0 dB
Without graph learning	0.113	66.17±0.24	44.87±0.22
Identity graph	0.335	67.81±0.28	46.51±0.24
Chain only	0.335	67.87±0.14	46.57±0.27
Similarity only	0.335	67.84±0.09	46.57±0.23
No temporal penalty	0.335	67.93±0.13	46.62±0.27
Full temporal similarity graph	0.335	**67.97** ±0.23	46.79±0.19

## Data Availability

The publicly available RadioML2016.10A, RadioML2016.10B, RML22, and RF Challenge datasets were used in this study. The original contributions presented in this study are included in the article. Further inquiries can be directed to the corresponding author.
